# Rare genetic susceptibility variants assessment in autism spectrum disorder: detection rate and practical use

**DOI:** 10.1038/s41398-020-0760-7

**Published:** 2020-02-24

**Authors:** Thomas Husson, François Lecoquierre, Kevin Cassinari, Camille Charbonnier, Olivier Quenez, Alice Goldenberg, Anne-Marie Guerrot, Anne-Claire Richard, Valérie Drouin-Garraud, Anne-Claire Brehin, Maryam Soleimani, Romain Taton, Maud Rotharmel, Antoine Rosier, Pascal Chambon, Nathalie Le Meur, Géraldine Joly-Helas, Pascale Saugier-Veber, Anne Boland, Jean-François Deleuze, Robert Olaso, Thierry Frebourg, Gael Nicolas, Olivier Guillin, Dominique Campion

**Affiliations:** 1 Normandie Univ, UNIROUEN, Inserm U1245 and Rouen University Hospital, Department of Genetics and Reference Center for Developmental Disorders, F 76000, Normandy Center for Genomic and Personalized Medicine, Rouen, France; 2 Department of Research, Centre hospitalier du Rouvray, Sotteville-Lès-Rouen, France; 3 Centre Ressources Autisme Normandie Seine Eure, Centre hospitalier du Rouvray, Sotteville-Lès-Rouen, France; 4 Centre National de Recherche en Génomique Humaine (CNRGH), Institut de Biologie François Jacob, CEA, Université Paris-Saclay, F-91057 Evry, France

**Keywords:** Autism spectrum disorders, Clinical genetics

## Abstract

Autism spectrum disorder (ASD) is a neurodevelopmental disorder with a strong genetic component whose knowledge evolves quickly. Next-generation sequencing is the only effective technology to deal with the high genetic heterogeneity of ASD in a clinical setting. However, rigorous criteria to classify rare genetic variants conferring ASD susceptibility are currently lacking. We have performed whole-exome sequencing to identify both nucleotide variants and copy number variants (CNVs) in 253 ASD patients, including 68 patients with intellectual disability (ID) and 90 diagnosed as Asperger syndrome. Using explicit criteria to classify both susceptibility genes and susceptibility variants we prioritized 217 genes belonging to the following categories: syndromic genes, genes with an excess of de novo protein truncating variants and genes targeted by rare CNVs. We obtained a susceptibility variant detection rate of 19.7% (95% CI: [15–25.2%]). The rate for CNVs was 7.1% (95% CI: [4.3–11%]) and 12.6% (95% CI: [8.8–17.4%]) for nucleotide variants. The highest rate (30.1%, 95% CI: [20.2–43.2%]) was obtained in the ASD + ID subgroup. A strong contributor for at risk nucleotide variants was the recently identified set of genes (*n* = 81) harboring an excess of de novo protein truncating variants. Since there is currently no evidence that the genes targeted here are *necessary and sufficient* to cause ASD, we recommend to avoid the term “causative of ASD” when delivering the information about a variant to a family and to use instead the term “genetic susceptibility factor contributing to ASD”.

## Introduction

Autism spectrum disorder (ASD), whose prevalence is estimated to be around 1 per 100 children, encompass a wide range of phenotypic manifestations ranging from severe behavioral impairment, often associated with intellectual disability (ID), to mild difficulties in social interaction. The fact that, clinically, autism is not a single disease entity is mirrored by the extreme genetic heterogeneity underlying this condition. Chromosomal microarray analysis is usually used as the first-tier genetic test for individuals with autism with a diagnostic yield of 7–9%^1,2^. However, such analysis cannot detect single nucleotide variations and small Indels but only loss or gain of genomic DNA material. In light of recent advances in ASD genetics, especially regarding the importance of de novo truncating variants, it is estimated that rare variants within several hundreds of genes may contribute to ASD susceptibility, each of them being present only in a very small proportion of cases^3^. In this context, next-generation sequencing is the only reasonable and cost-effective approach to search for variants in these genes in a diagnostic perspective.

The aims of the present study were threefold: (i) to establish an accurate list of susceptibility genes from literature data and to provide guidelines to categorize rare susceptibility variants (ii) based on these criteria, to estimate the whole exome sequencing (WES) detection rate of rare susceptibility variants in a sample of ASD patients typical of those attending genetic consultations and (iii) to propose some recommendations for susceptibility factors assessment and interpretation in clinical practice.

## Methods

### Patient recruitment

During the 2009–2017 period, 679 unrelated subjects received a diagnosis of ASD or Asperger syndrome by clinicians of our local expert center. This study was carried out on a subset of 253 cases, including all patients from whom parental DNA was available (*n* = 159) and 94 randomly selected patients.

Clinical evaluation was mainly based on ADOS-2. In addition, ADI-R and CARS, were also used for 76 and 145 patients, respectively. All diagnoses were made according to DSM-IV-TR criteria. Accordingly, the diagnosis of Asperger syndrome included the absence of language impairment, as documented during clinical examination. IQ was assessed in subjects with age >5 years with the Wechsler Intelligence Scale for Children (WISC) or the Raven Progressive matrix. PsychoEducational Profil third edition (PEP-3) and Vineland Adaptive Behavior Scales (VABS-2) assessment, when available, were used to refine the classification of cases with heterogeneous cognitive functions or mild cognitive impairment. Subjects with age <5 years were classified in the ASD subgroup, without further specification.

Following national recommendations, diagnostic assessment included a genetic consultation. Every patient was received by a senior clinical geneticist for thorough questioning about development, previous medical records and family history, followed by a complete physical examination.

This genetic study was approved by our legal ethics committee. Parents or legal representative of the children signed an informed consent for genetic analyses.

### Molecular genetics

All included patients had previously been negatively screened for Fragile X expansion and 54 had been tested by array Comparative Genomic Hybridization (aCGH) Agilent 180K (Santa Clara, United-States), yielding to the prior identification (subsequently confirmed by exome sequencing) of four CNVs conferring susceptibility for ASD. For each proband, DNA was extracted from fresh blood samples. DNA was also obtained from affected siblings and from parents when available.

Exome sequencing was performed on every proband. Exomes were captured using the Agilent SureSelect All Exons V5-UTR or V6 Kit (Santa Clara, United-States). Sequencing was performed on an Illumina HiSeq4000 (Illumina, San Diego, CA, USA) at the CNRGH (*Centre National de Recherche en Génomique Humaine, Evry, France)* with paired end mode, 150 base pairs (bp) reads. Nucleotide variants and copy number variants (CNVs) were analyzed through a bioinformatics pipeline to perform variant calling, quality check, annotation and CNVs detection (see Sup Info). When possible, the segregation of retained variants was examined by Sanger sequencing for nucleotide variants and by ddPCR or QMPSF for CNVs. Parenthood was checked by polymorphic microsatellites for patients carrying de novo mutations.

### List of genes and interpretation of variants

To establish a panel of genes firmly contributing to ASD susceptibility, we retained three sets of genes:

(i) genes showing a statistically significant excess of de novo protein truncating variants (PTVs), (*i.e*. nonsense, canonical splice site variants and frameshift indels) in large published cohorts. Since these genes have been identified in several, sometimes redundant recent trio studies^1,4–10^, we reanalyzed (see Sup Info) all de novo events reported in each of these studies (Sup Table 1). Depending on the level of statistical evidence for the enrichment in de novo PTVs, we ranked these genes into two categories: class A (false discovery rate (FDR) <5%) meaning definitely involved and class B (5% ≤ FDR ≤ 10%), meaning probably involved in ASD susceptibility. This led to a list of 81 genes (Sup Table 2). For these genes, we retained as susceptibility factor all rare PTVs found in patients, whatever the inheritance (i.e. de novo occurrence or not).

(ii) syndromic ASD–related genes, i.e. genes causing monogenic diseases for which ASD can be part of the phenotype (*n* = 107). This set of genes was carefully curated from the Simon Foundation Autism Research Initiative (Sfari) database and from literature data. For each syndromic gene, the mode of inheritance was specified and rare non-synonymous variants were prioritized. For these genes, pathogenicity of rare variants (MAF < 1% in GnomAD) was evaluated using the guidelines from the American College of Medical Genetics-Association for Molecular Pathology (ACMG-AMP). Only variants considered as likely pathogenic or pathogenic (class 4 or 5) were retained as advised by the guidelines. For our analysis, we considered these variants as probable (class 4) or definite (class 5) ASD-susceptibility variants and then evaluated whether patients carrying them harbored or not the full syndromic presentation typical of each gene.

(iii) genes intersected by a burden of rare CNVs, listed in the Sfari database (*n* = 85).

**Fig. 1:**
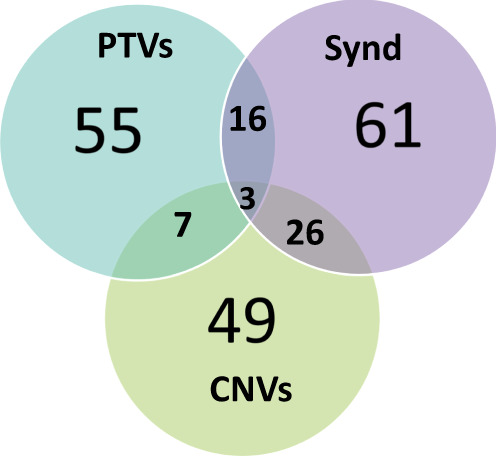
Venn diagram of the gene list stratified in three categories: syndromic genes, genes with an excess of de novo PTVs, genes intersected by a burden of rare CNVs.

Combining these three sets yielded a high confidence list of 217 ASD susceptibility genes (Sup Table 3). Note that some of these genes were simultaneously present in several sets. (Fig. 1).

Besides this main panel and despite the fact that the interpretation was more problematic, we also examined rare missense variants in genes carrying a burden of de novo and transmitted mutations identified by TADA analyses^4,11^ as well as in genes recently reported as carrying an excess of de novo missense mutations in ASD and developmental disorders (DD)^1,12^. This analysis was conducted on 89 genes (61 already included in the main list and 28 additional genes). For this analysis, only de novo missense variants predicted as damaging by three in silico prediction tools were considered.

Finally, for patients with ID or additional sensory impairment, we examined all OMIM referenced genes.

While CNV detection was performed on all genes of the main list, variant interpretation depended on the gene subsets. We retained all previously associated CNVs (i.e. duplications or deletions) within the set of large recurrent CNVs associated with various DDs including ASD^13^. In contrast, we used a conservative approach to interpret the remaining CNVs, and only retained rare deletions targeting Loss of Function (LoF) intolerant genes as probable ASD susceptibility variants.

## Results

Among the 253 ASD probands (81.4% males), 68 were categorized in the ASD+ID subgroup while 90 were classified as having Asperger syndrome. The average age at inclusion was 8.5 ± 5.6 years (range 3–41). Positive family history (among first, second or third-degree relatives) for ASD, DD or ID was reported for 27.7% of probands. Demographic and phenotypic characteristics of the sample are displayed in Table 1.

**Table 1:** Demographic and phenotypic characteristics of the sample.

	% (N/253)
Males	81.4% (206)
Family history	27.7% (70)
Consanguinity	1.6 % (4)
Asperger syndrome	35.6% (90)
Intellectual disability	26.9% (68)
Dysmorphic features	26.5% (67)
Premature birth	3.95% (10)
Microcephaly	3.7% (9)
Macrocephaly	7.9% (20)
Epilepsy	7.1% (18)

As shown in Table 2, ASD-contributing CNVs were present in 18/253 cases (7.1%, 95% CI: [4.3–11%]) while ASD-contributing nucleotide variants (Table 3) were detected in 32/253 subjects (12.6%, 95% CI: [8.8–17.4%]), yielding to a global detection rate of 50/253 (19.7%, 95% CI: [15–25.2%]). Of note, no patient carried multiple susceptibility variants. A slightly higher detection rate (29.8%, 95% CI: [17.3–44.9%]) was found in females. When the sample was split into three clinical subgroups, the rate of CNVs and nucleotide susceptibility-variants was particularly high in the ASD+ID subgroup (30.1%, 95% CI: [20.2–43.2%]) (Table 4). In the whole sample, two CNVs (*NRXN1* E1–3 del and 15q11 BP 1–2 del) were recurrent and four genes were found to carry nucleotide variants more than once: *CHD8, SHANK3, NRXN1*, and *KMT2A*. Regarding nucleotide susceptibility variants, the contribution of the set of genes previously shown as enriched in de novo PTVs in large ASD cohorts should be highlighted. Ten genes carrying ASD-susceptibility variants belonged to this category vs seven syndromic genes and nine genes belonging simultaneously to the two categories. Overall, the high frequency of de novo events is noteworthy: we identified 21/28 de novo nucleotide variants and 6/14 de novo CNVs among 42 patients from whom parental DNA was available. Regarding the mode of inheritance of ASD contributing variants, no autosomal recessive inheritance was encountered in this mainly outbred population.

**Table 2:** CNVs conferring susceptibility for autism identified among the 253 ASD subjects.

ID	Chr	Start (hg19)	End (hg19)	Size (bp)	Type	Syndrome/gene	Inheritance	Diag	Age	Sex	Family history
*Pathogenic CNVs*											
266	15	22833359	23330473	497114	Del	15q11 del syndrome (BP1-BP2)	Maternal	Asperger	10	M	S
175	15	22833359	23330473	497114	Del	15q11 del syndrome (BP1-BP2)	Maternal	ASD	6	M	S
298	15	22833359	23330473	497114	Del	15q11 del syndrome (BP1-BP2)	NA	Asperger	13	F	S
277	15	22833359	23330473	497114	Del	15q11 del syndrome (BP1-BP2)	NA	Asperger	16	M	Fa
207	15	23686022	28567298	4881276	Dup	15q11.2q13 dup syndrome (BP2-BP3)	DN	ASD	9	M	Fa
405	15	23686022	30114707	6428685	Dup	15q11.2q13 dup syndrome (BP2-BP4)^c^	DN	ASD	5	F	S
225	16	14968893	16267261	1298368	Del	16p13 del syndrome	Maternal	ASD+ID	5	F	S
106	16	14968893	16267261	1298368	Dup	16p13 dup syndrome	Paternal	ASD+ID	11	F	S
40	16	29674271	30198544	524273	Dup	16p11.2 dup syndrome	DN	Asperger	11	M	Fa
392	16	29674271	30198544	524273	Del	16p11.2 del syndrome	DN	ASD+ID	7	M	S
288	22	51123001	51220846	97845	Del	SHANK3 (E9–22)+2 other genes	DN	ASD+ID	32	M	S
394	X	144899347	148678216	3778869	Del	Del Xq27.3-q28 (15 genes including FMR1 and AFF2)^c^	NA	ASD+ID	24	F	Fa
52	1	146500897	147806796	1305899	Dup	1q21.1 dup syndrome	Not maternal	ASD	8	M	S
304	2	51253508	51259674	6166	Del	NRXN1 (E1–3)^c^	Paternal	ASD+ID	6	M	Fa
396	2	51253508	51259674	6166	Del	NRXN1 (E1–3)^c^	DN	ASD+ID	7	M	S
318	2	51253508	51259674	6166	Del	NRXN1 (E1–3)	Paternal	Asperger	12	M	S
*Likely pathogenic CNVs*											
55	18	44624417	44641759	17342	Del	KATNAL2 (E 16–18)	Paternal	ASD	6	M	Fa^a^
365	9	119373136	119495896	122760	Del	ASTN2 (E1–4)	Maternal	Asperger	19	F	Fa^b^

*NA* not assessed, *E* exon, *BP* breakpoint, *DN* de novo, *Del* deletion, *Dup* duplication, *F* female, *M* male, *S* sporadic, *Fa* familial.

^a^1^st^Sib (ASD): wild type and 2^nd^ Sib (language delay): variant carrier.

^b^Sib (Asperger): variant carrier.

^c^Already identified by aCGH.

**Table 3:** Nucleotide variants conferring susceptibility for autism (stratified by category) identified among the 253 ASD subjects. Nucleotide variants in genes harboring a statistically significant excess of de novo PTVs were classified either as susceptibility variants in class A genes (false discovery rate (FDR) <5%) or as probable susceptibility variants in class B genes (10% < FDR < 5%). Nucleotide variants in syndromic ASD–related genes were classified using the guidelines from the American College of Medical Genetics-Association for Molecular Pathology.

ID	Gene	Variation	Transcript	Inheritance	o/e	GnAD	Diag	Age	Sex	Family history
(a) Nucleotide variants in genes harboring a statistically significant excess of de novo PTVs										
*Susceptibility variants*										
213	*CHD8*	p.Gln1171* c.3511C>T	NM_001170629.1	Paternal	3.94%	_	ASD+ID	7	F	S
223	*CHD8*	p.Lys528Glyfs*14 c.1582_1583delAA	NM_001170629.1	DN	3.94%	_	Asperger	19	M	S
220	*CHD8*	p.Leu980Argfs*5 c.2937_2939delinsTC	NM_001170629.1	DN	3.94%	_	Asperger	30	M	S
98	*NRXN1*	p.Leu158Alafs*29 c.471dupG	NM_001135659.1	DN	12.30%	1/242 430	Asperger	12	M	S
307	*NRXN1*	p.Glu389Serfs*3 c.1165delG	NM_001135659.1	Maternal	12.30%	_	ASD	3	M	S
253	*ANK2*	p.Glu3062* c.9184G>T	NM_001148.4	DN	6.20%	_	Asperger	11	M	S
325	*GIGYF1*	p.Ter1036Glyext*72 c.3106T>G	NM_022574.4	DN	35.64%	_	ASD	12	M	Fa^a^
341	*KDM5B*	p.Arg1397* c.4189C>T	NM_006618.4	NA	44.95%	2/247 406	ASD	4	M	S
*Probable susceptibility variants*										
122	*RIMS1*	p. ? c.100+1G>A	NM_001168407.1	Maternal	18.65%	1/31 404	ASD+ID	14	F	S
188	*SETD2*	p.Arg1708* c.5122C>T	NM_014159.6	DN	13%	_	Asperger	8	F	Fa^b^
301	*DIP2A*	p. ? c.1429+2T>G	NM_015151.3	Maternal	23.89%	_	Asperger	6	F	S
285	*HECTD4*	p.Ala3108Glyfs*5 c.9322dupG	NM_001109662.3	Maternal	7.56%	_	Asperger	12	M	S
168	*SPEN*	p.Ser2411* c.7232C>A	NM_015001.2	DN	2.31%	_	Asperger	6	M	S

(b) Nucleotide variants in syndromic ASD-related genes										
*Susceptibility variants*										
370	*EBF3*	p.Gln78* c.232C>T	NM_001005463.2	DN	3%	_	ASD	4	M	S
79	*TSC2*	p.Val1062Glyfs*7 c.3185_3188del	NM_000548.3	DN	2%	_	ASD+ID	8	F	S
*Probable susceptibility variants*										
30	*DEAF1*	p.Arg224Gln c.671G>A^c^	NM_021008.3	Maternal	41%	1/246 022	ASD+ID	14	M	S
328	*DNMT3A*	p.Gln249* c.745C>T	NM_022552.4	DN	126%	1/244 420	ASD+ID	10	M	S
215	*AUTS2*	p.Thr328Argfs*19 c.983_984del	NM_015570.3	DN	13%	_	ASD+ID	4	M	S
222	*DYNC1H1*	p.? c.4396–1G>C	NM_001376.4	DN	5%	_	ASD	27	F	Fa^a^
121	*NR2F1*	p.Gly395Ala c.1184G>C	NM_005654.5	DN	0.00%	_	ASD+ID	20	M	S
(c) Nucleotide variants in genes belonging to both categories										
*Susceptibility variants*										
296	*SHANK3*	p.Pro922Argfs*34 c.2765del	NM_033517.1	DN	3.98%	_	ASD+ID	8	F	S
377	*SHANK3*	p.Leu1030Cysfs*48 c.3088delC	NM_033517.1	DN	3.98%	_	ASD+ID	6	M	S
353	*SHANK3*	p.Ala1227Glyfs*69 c.3679dupG^d^	NM_033517.1	NA	3.98%	_	ASD+ID	31	F	S
136	*GRIN2B*	p.Ser9Phefs*50 c.23_24insC	NM_000834.3	DN	0.00%	_	ASD	4	M	S
85	*ASXL3*	p.Ser1246* c.3737C>A	NM_030632.1	DN	9.86%	_	ASD+ID	11	M	Fa
78	*SCN2A*	p.Phe601Leufs*40 c.1800delC	NM_001040142.1	DN	6.33%	_	ASD+ID	12	F	S
382	*CHD2*	p.Val332Glyfs*25 c.995_999delTGAAG	NM_001271.3	NA	2.75%	_	ASD+ID	7	M	S
47	*DYRK1A*	p.Arg255* c.763C>T^e^	NM_101395.2	Not maternal	8.45%	_	ASD+ID	18	M	S
395	*ANKRD11*	p.Glu1154Glyfs*16 c.3460dup	NM_001256182.1	DN	4.72%	_	Asperger	11	M	S
*Probable susceptibility variants*										
291	*KMT2A*	p. ? c.10835+1G>A^f^	NM_001197104	Paternal	3.12%	_	Asperger	6	M	S
324	*KMT2A*	p.Gln3192Pro c.9575A>C	NM_001197104	DN	3.12%	_	ASD	9	M	S
157	*MECP2*	p.Glu282Gly c.845A>G	NM _004992.3	DN	10.72%	_	ASD	13	F	S

*F* female, *M* male, *S* sporadic, *Fa* familial, *DN* de novo, *NA* not assessed, *o/e* observed/expected ratio of PTVs in GnomAD, *GnAD* allelic frequency in the GnomAD database.

^a^Sib (ASD): wild type.

^b^Sib (Asperger): wild type.

^c^p.Arg224Trp found as a de novo event in an ASD case^27^.

^d^Variation found as de novo event in ASD+ID cases^28^.

^e^Variation found as a de novo event in a syndromic case of DYRK1A-haploinsufficiency^29^.

^f^Variation found in three related ID patients^14.^.

**Table 4:** Detection rate for CNVs and nucleotide variants among the 253 ASD subjects stratified by clinical subgroups.

	ASD+ID *n* = 68	ASD *n* = 95	Asperger *n* = 90	Total *n* = 253
CNVs	7	5	6	18–7.1% [4.3–11%]
Nucleotide variants	14	8	10	32–12.6% [8.8–17.4%]
Total	21–30.1% [20.2–43.2%]	13–13.7% [7.5–22.3%]	16–17.7% [10.5–27.3%]	50–19.7% [15–25.2%]

Finally, variants in two genes not included in our list, but which cause nevertheless part of the phenotype, were found in two patients presenting ASD, ID, and sensory deficits. A patient with hearing impairment carried compound heterozygous c.2485C > T, p.Gln829*/c.5665T > C, p.Trp1889Arg (NM_194248.2) *OTOF* (MIM: 601071) pathogenic variants, while a patient with severe visual impairment, born from consanguineous parents, carried a homozygous c.389del, p.Pro130Leu fs*36 (NM_000180.3) *GUCY2D* (MIM: 204000) pathogenic variant. These mutations explain the sensory deficits of these patients but there is currently no evidence that they are related to the ASD phenotype.

### Genotype/phenotype correlations

Consistency with the previously described phenotypes was checked for syndromic genes and was fair to good for most genes (*DNMT3A, ASXL3, EBF3, NR2F1, DYRK1A, DEAF1, ANKRD11, SHANK3*). (See Sup Info) but in some cases unusual phenotypes were encountered:

Patients carrying *MECP2* or *KMT2A* mutations did not harbor the classical features of Rett or Wiedemann-Steiner syndrome, respectively. A *MECP2* mutation (not present in the International Rett database) was identified in a girl who presented with mild ASD. She had no developmental delay (walk at 15 months, no language delay) and was enrolled in an ordinary school at the age of 14. She demonstrated no ID and no dysmorphic features. *KMT2A* mutations were identified in two patients. One of them carrying the p.Gln3192Pro de novo mutation was the first child of a couple with epilepsy and mild cognitive impairment. He had a global developmental delay with a sitting age at 1 year but a normal walking age at 17 months, a language delay and a motor instability. Clinical evaluation showed a normal growth, an hyperlaxity and a diffuse hypertrichosis but no hairy elbow or morphological features typical of Wiedemann–Steiner Syndrome. The second one was the first child of a couple without medical history. He carried a c.10835 + 1G > A splice mutation already described in 3 related ID patients, not diagnosed as Wiedemann-Steiner. This variant leads to the in frame deletion of exon 28 and likely disrupts the stabilizing interaction between the C terminal KMT2A fragments^14^. Our patient had no motor delay but a language delay. Electroencephalogram showed left occipital spikes, but he never had seizures. MRI showed an isolated agenesis of the corpus callosum. The diagnosis of Wiedemann-Steiner syndrome was not clinically evoked.

Likewise, although *DYNC1H1* missense or truncating variations are usually associated with a severe phenotype including ID, neuronal migration defects and epileptic encephalopathy^15^, our patient had an unremarkable ASD phenotype without neurological features. *CHD2* truncating mutations are mostly associated with pediatric refractory epilepsy^16^, but our patient had only experienced one tonic-clonic seizure. In the same vein and in line with recent reports underlining the phenotypic heterogeneity of *SCN2A*^17^ and *GRIN2B*^18^ mutation carriers, none of the patient carrying de novo truncating mutations on these genes had epilepsy, which is a feature generally associated with these mutations.

It should also be noted that besides these well-characterized syndromic genes, another patient carrying the *RIMS1* splice variant received a diagnosis of Dravet syndrome but bore no mutation on genes causing this phenotype. Interestingly, the RIM1a protein encoded by the mice ortholog of *RIMS1* has been characterized as a presynaptic active zone protein mainly expressed in neurons from the cerebellum^19^ and controlling epileptogenesis following pharmacologically induced status epilepticus^20^. This suggests that the *RIMS1* truncating variant identified here, which affects a minor transcript expressed in the cerebellum (https://gtexportal.org), could be associated with a peculiar neurological phenotype.

## Discussion

In this report, the rate of identified CNVs, largely based on well-known recurrent CNVs, was similar to that obtained in several recent studies^1,2^. It is notable that four of our patients carry the 15q11.2 recurrent deletion which is known to have a mild effect on developmental disorder. Hence, it is very probable that additional factors are involved in this subset. Regarding nucleotide variants, we used rigorous criteria to classify both ASD susceptibility genes and variants. As a result, we retained only 217 genes as compared for example with the 880 genes present in the Sfari database (october 2018 release), often with minimal evidence of involvement in ASD susceptibility. We nevertheless obtained a detection rate of 12.6% for nucleotide variants. Combining CNVs and nucleotide variants, we reached a higher detection rate (19.7%) than the one obtained in 105 ASD patients previously analyzed by WES and aCGH (15.8%)^2^. This is partly due to the contribution of the set of genes with an excess of de novo PTVs in ASD patients, which were identified in the recent years thanks to the study of more than 6000 ASD trios^1,4–10^. Clearly, this huge effort has obvious consequences for clinical practice. The finding of a particularly high rate of de novo PTVs in this set of genes (14/20 PTVs = 70% of the total number of PTVs in patients for whom parental DNA was available) reinforces the conclusion that this set is truly enriched in de novo PTVs in ASD subjects. As seen in Sup Table 2, the gene which is the most frequently hit by de novo PTVs in ASD patients from the literature is *CHD8*^21^. Interestingly, among the three *CHD8* PTVs identified in this study, two were de novo events while the third was inherited from a parent whose sole phenotypic feature was macrocephaly. More generally, the presence of 30% of PTVs inherited from parents with no ASD diagnosis underlines the incomplete penetrance and/or variable expressivity of these variants.

Due to the stringent criteria used to classify variants, it should be stressed that our detection rate is likely to be underestimated. First, concerning CNVs, partial duplications intersecting genes were conservatively considered of uncertain significance (Sup Table 4). However, it is well known that tandem intragenic duplications often disrupt reading frames and can lead to haploinsufficiency. In a recent analysis of all structural variation detected in the gnomAD dataset, the authors showed a significant correlation between constraint metrics reflecting intolerance to Lof mutations and the depletion of partial genic duplications at the gene level^22^. However, despite these findings, the individual interpretation of partial duplications remains challenging and will require additional investigations. Second, since all missense variants impacting genes exhibiting a statistically significant excess of de novo missense mutations in ASD^1,12^ were inherited, we decided to consider them as variants of unknown significance (VUS) (Sup Table 5) even when they were absent from gnomAD.

The detection rate was particularly high in the ASD+ID subgroup. It was in the same range as those recently reported in ID or DD patients (25–39%)^23–25^, or in a cohort of 163 ID/DD patients “with reported ASD or autistic features” (25.8%)^26^. Of note, all these studies used larger panels of genes than ours. In our study, although ASD+ID patients were more likely hit, susceptibility variants were also found with a high frequency in the Asperger group. Several genes carried susceptibility variants in both ASD+ID and Asperger subjects. For example, in accordance with a previous report outlining that cognitive ability of *CHD8* patients ranges from profoundly disabled to age-appropriate^21^, of the three patients carrying *CHD8* PTVs, one had ID associated to ASD while two others received a diagnosis of Asperger syndrome. However, macrocephaly, a shared feature among *CHD8* patients, was present in all three patients. Likewise, *NRXN1* exon 1–3 deletions were present in patients with each clinical presentation (ASD, ASD+ID, Asperger).

The genotype/phenotype correlation for syndromic genes was generally good, but in some cases was not typical. As already outlined^23,25^, it is noteworthy that the phenotypic spectrum of mutations in several “syndromic genes” for which a specific phenotype has previously been described is broader than initially thought. Clearly, for a large set of genes, prioritizing by double entries (genes with both an excess of de novo PTVs and syndromic genes) allows the identification of ASD susceptibility factors in more patients than prioritizing upon a single criteria based on strict phenotypic concordance with described syndromic features.

In summary, the strategy we advocate here is an efficient and cost-effective manner to deal with the detection of both rare nucleotide variants and CNVs in a clinical setting. Of course, exome data should be periodically reanalyzed and the list of genes updated. Due to the extreme rarity of variants studied here, it is currently difficult to establish for each of them if they are fully causative of an ASD phenotype. Most likely, only a subset of them are *necessary and sufficient* to cause ASD. For this reason, we recommend to avoid the term “causative of ASD” when delivering the information about these variants to a family and to use instead the term “genetic susceptibility factor contributing to ASD phenotype”. At the present stage, with the exception of pathogenic variants in syndromic genes causing well-defined monogenic diseases, using these results for prenatal diagnosis is premature.

## Supplementary information

Supplementary information

Table S1. De novo PTVs among ASD patients from SSC, ASC, ACGC, TASC, MSSNG and AGRE cohorts.

Table S2. List of gene with a significant excess of de novo truncating variants in the literature.

Table S3. List of 217 genes conferring ASD susceptibility prioritized in this study.

Table S4. CNVs of unknown significance found among the 253 ASD subjects.

Table S5. Nucleotide variants of unknown significance found among the 253 ASD subjects.
