# Supplementary material for: Rare genetic susceptibility variants assessment in autism spectrum disorder: detection rate and practical use
Source: Transl Psychiatry. 2020 Feb 24;10:77. doi: 10.1038/s41398-020-0760-7 (PMC7039996; doi:10.1038/s41398-020-0760-7)
Supplement: Supplementary file 1 — Supplementary information [file 41398_2020_760_MOESM1_ESM.docx]

**Supplementary information**

**Exome sequencing: bioinformatics pipeline**

**List of genes with an excess of *de novo* truncating mutations**

**Statistical Analysis**

**Consistent genotype/phenotype correlations for syndromic genes**

**References**

**Supplementary tables**

**Exome sequencing: bioinformatics pipeline**

All exome samples were processed through the same bioinformatics pipeline following the Best Practices Guidelines from the Broad Institute as previously described ^1^. WES resulted in an average individual median depth of coverage on targeted exome kits of 102x, with 95% of individual median depths between 49x and 169x. Reads were mapped to the GRCh37 1000Genomes build using BWA 0.7.5a ^2^. Picard Tools 1.101 (http://picard.sourceforge.net) was used to flag duplicate reads. GATK 3.3 ^3^ was applied for short insertion and deletion (InDels) realignment, base quality score recalibration (BQSR) and finally single nucleotide variants and InDel discovery using Haplotype Caller across all samples simultaneously. A quality score (VQSLOD) was estimated for each variant with the VQSR function from GATK. All genotypes satisfying the following quality filters were considered candidates for analysis and manually reviewed prior to Sanger sequencing validation: genotype read depth > 6 and genotype quality > 20. Variants were annotated with SnpEff 4.2 ^4^ and SnpSift 4.2 ^4^ software using dbNSFP 2.9.1 and Ensembl GRCh37.75. Variants with a minor allele frequency (MAF) of <1% in the gnomAD database ^5^ (http://gnomad.broadinstitute.org/) located in exonic or canonical splice site regions (± 2 bp around each coding exon) were then extracted within every exon from the list of genes.

In silico prediction of pathogenicity of missense variants was assessed by Polyphen-2 (HumDiv) ^6^, SIFT ^7^ and MutationTaster2 ^8^.

*CNV analysis*

As previously described ^9^ CNVs were detected from exome data using CANOES software ^10^, an algorithm dedicated to the detection of quantitative genomic variations based on read depth information. CNVs detected by CANOES were then validated by Digital Droplet PCR (ddPCR), QMPSF or aCGH.

**List of genes with an excess of *de novo* truncating mutations**

We consulted the denovo-db database ^11^ and we directly reviewed the raw data of published studies reporting *de novo* mutations in ASD patients from the SSC (2508 trios ^12–14^), ASC (1445 trios ^15^) and MSSNG (1627 trios ^16^) dataset. We also took into account studies focusing on gene panels in three datasets: AGRE dataset (1732 trios) ^17^), ACGC dataset (1045 trios) ^18^ and TASC dataset ( 921 trios) ^19^. Thus, according to the gene under study, the number of reported trios ranged from 5580 to 9279. Among these trios we compiled 865 *de novo* PTVs (Sup Table 1). To evaluate gene-specific enrichment in *de novo* PTVs in this sample we performed simulations redistributing the observed number of mutations at random over all genes based on their relative mutation rate π_g_. More precisely, we derived gene-specific *relative* mutation probabilities from gene-specific expected counts of loss of function (LoF) single nucleotide variations *(*nonsense + canonical splicing) provided by the gnomAD browser. Only 804 *de novo* PTVs that fell on an autosomal gene with mutation rate information were included in the analysis. To extend gnomAD constraint analysis to indels, we assumed that the relative occurrence of single nucleotide variations and indels did not vary across genes. To account for the inclusion of gene panels, we weighted gene-specific expected counts according to the number of samples sequenced for each gene. Weighted expected counts were then divided by the sum of all weighted expected counts to obtain a probability distribution. In short, if e_g_ denotes the expected PTV count for gene g given by gnomAD, and N_g_ the total number of samples with information on gene g we compute:

π_g_ = e_g_* N_g_ / Σ(e_g_ * N_g_)

For a given gene g, π_g_ represents the probability that one observed *de novo* mutation should hit gene g instead of any other gene, taking into account both its relative mutability and its coverage by exomes and gene panels. We simulated 1,000,000 samples redistributing the 804 *de novo* PTVS over all genes according to this multinomial distribution. Gene-specific p-values for enrichment were then computed as the proportion of simulations in which the number of mutations hitting a gene was the same or exceeded the observed number of *de novo* mutations. P-values were adjusted for multiple testing using the Benjamin-Hochberg FDR correction. All analyses were performed with the R statistical software ^20^ (https://www.R-project.org/).

Overall, an excess of *de novo* PTVs was found for 42 genes with a false discovery rate (FDR) < 5% and 82 with a FDR ≤10% (Sup Table 2). Most of these genes were highly intolerant to loss of function (LoF) as evidenced by a low o/e ratio, with a cut-off at 0·35 on the upper-bound of the o/e confidence interval (LOEUF, Loss-of-function Observed/Expected Upper bound Fraction) as advised by gnomAD v2.a for use in clinical interpretation of Mendelian cases. Three genes were discarded from the list because of an excessive o/e ratio (LOEUF>1), denoting high tolerance to LoF mutations. Interestingly, 14 genes displayed an intermediate o/e ratio (0·35<LOEUF<1). Some of them are considered as ASD-contributing with a high level of evidence (e.g. *PTEN*). Hence, not expecting a full penetrance regarding ASD for all of them, we kept these genes in our list. On the other hand, we added two X linked genes (*MECP2* and *UPF3B*) for which it was not possible to quantify the extent of the enrichment without gender information from all sequenced individuals, thus leading to a final list of 81 genes.

**Statistical Analysis**

The 95% CI of the detection rates were calculated based on a binomial distribution.

**Consistent genotype/ phenotype correlations for syndromic genes**

-The phenotype of the patient carrying the *DNMT3A* *de novo* truncating mutation was highly suggestive of Tatton-Brown-Rahman syndrome ^21^ and associated increased prenatal and post-natal growth, umbilical hernia, and ID.

-The patient carrying the *ASXL3* *de novo* truncating mutation presented with severe ID, speech deficiency, hyperlaxity, feeding difficulties, facial features consistent with a diagnosis of Bainbridge Ropers syndrome ^22^.

-The patient with the *EBF3* *de novo* truncating mutation exhibited ID associated with cerebellar ataxia, congenital hypotonia and strabismus, consistent with previous reports ^23^.

-Features of the Bosh-Boonstra-Schaaf syndrome (ID, seizures, visual impairment) ^24^ were present in the patient with the *NR2F1 de novo* missense mutation.

-The patient carrying the p.Arg255* *DYRK1A* truncating mutation – already identified in a recent report as a *de novo* event in one ASD case ^25^ – harbored the classical phenotypic profile of *DYRK1A* haploinsufficiency, including microcephaly, severe ID, facial dysmorphic features and visual impairment with optic atrophy.

-The *DEAF1* missense variant p.Arg224Gln was found in a patient with severe ID and near complete absence of language. It occurred in the functionally important SAND domain of the protein at a position where another DN substitution (p. Arg224Trp) has already been described in a subject with ASD+ID ^26^. It was however inherited from an asymptomatic parent when all previously reported pathogenic *DEAF1* mutations occurred *de novo.*

-The clinical features of KBG syndrome (brachycephaly, macrodontia, brachydactyly, short stature) ^27^ were present in the patient carrying the *ANKRD11* mutation.

**References**

1. Bellenguez, C. *et al.* Contribution to Alzheimer’s disease risk of rare variants in TREM2, SORL1, and ABCA7 in 1779 cases and 1273 controls. *Neurobiol. Aging* **59**, 220.e1-220.e9 (2017).

2. Li, H. & Durbin, R. Fast and accurate short read alignment with Burrows-Wheeler transform. *Bioinformatics* **25**, 1754–1760 (2009).

3. McKenna, A. *et al.* The Genome Analysis Toolkit: a MapReduce framework for analyzing next-generation DNA sequencing data. *Genome Res.* **20**, 1297–1303 (2010).

4. Cingolani, P. *et al.* A program for annotating and predicting the effects of single nucleotide polymorphisms, SnpEff: SNPs in the genome of Drosophila melanogaster strain w1118; iso-2; iso-3. *Fly (Austin)* **6**, 80–92 (2012).

5. Lek, M. *et al.* Analysis of protein-coding genetic variation in 60,706 humans. *Nature* **536**, 285–291 (2016).

6. Adzhubei, I. A. *et al.* A method and server for predicting damaging missense mutations. *Nat. Methods* **7**, 248–249 (2010).

7. Ng, P. C. & Henikoff, S. Predicting deleterious amino acid substitutions. *Genome Res.* **11**, 863–874 (2001).

8. Schwarz, J. M., Cooper, D. N., Schuelke, M. & Seelow, D. MutationTaster2: mutation prediction for the deep-sequencing age. *Nat. Methods* **11**, 361–362 (2014).

9. Le Guennec, K. *et al.* 17q21.31 duplication causes prominent tau-related dementia with increased MAPT expression. *Mol. Psychiatry* **22**, 1119–1125 (2017).

10. Backenroth, D. *et al.* CANOES: detecting rare copy number variants from whole exome sequencing data. *Nucleic Acids Res.* **42**, e97 (2014).

11. Turner, T. N. *et al.* denovo-db: a compendium of human de novo variants. *Nucleic Acids Res.* **45**, D804–D811 (2017).

12. Dong, S. *et al.* De novo insertions and deletions of predominantly paternal origin are associated with autism spectrum disorder. *Cell Rep* **9**, 16–23 (2014).

13. Iossifov, I. *et al.* The contribution of de novo coding mutations to autism spectrum disorder. *Nature* **515**, 216–221 (2014).

14. Krumm, N. *et al.* Excess of rare, inherited truncating mutations in autism. *Nat. Genet.* **47**, 582–588 (2015).

15. De Rubeis, S. *et al.* Synaptic, transcriptional and chromatin genes disrupted in autism. *Nature* **515**, 209–215 (2014).

16. C Yuen, R. K. *et al.* Whole genome sequencing resource identifies 18 new candidate genes for autism spectrum disorder. *Nat. Neurosci.* **20**, 602–611 (2017).

17. Stessman, H. A. F. *et al.* Targeted sequencing identifies 91 neurodevelopmental disorder risk genes with autism and developmental disability biases. *Nat Genet* **49**, 515–526 (2017).

18. Wang, T. *et al.* De novo genic mutations among a Chinese autism spectrum disorder cohort. *Nat Commun* **7**, 13316 (2016).

19. O’Roak, B. J. *et al.* Recurrent de novo mutations implicate novel genes underlying simplex autism risk. *Nat Commun* **5**, 5595 (2014).

20. R Core Team. R: A language and environment for statistical computing. R Foundation for Statistical Computing. (2017).

21. Tatton-Brown, K. *et al.* Mutations in the DNA methyltransferase gene DNMT3A cause an overgrowth syndrome with intellectual disability. *Nat. Genet.* **46**, 385–388 (2014).

22. Balasubramanian, M. *et al.* Delineating the phenotypic spectrum of Bainbridge-Ropers syndrome: 12 new patients with de novo, heterozygous, loss-of-function mutations in ASXL3 and review of published literature. *J. Med. Genet.* **54**, 537–543 (2017).

23. Sleven, H. *et al.* De Novo Mutations in EBF3 Cause a Neurodevelopmental Syndrome. *Am. J. Hum. Genet.* **100**, 138–150 (2017).

24. Kaiwar, C. *et al.* Novel NR2F1 variants likely disrupt DNA binding: molecular modeling in two cases, review of published cases, genotype-phenotype correlation, and phenotypic expansion of the Bosch-Boonstra-Schaaf optic atrophy syndrome. *Cold Spring Harb Mol Case Stud* **3**, (2017).

25. Earl, R. K. *et al.* Clinical phenotype of ASD-associated DYRK1A haploinsufficiency. *Mol Autism* **8**, (2017).

26. Vulto-van Silfhout, A. T. *et al.* Mutations affecting the SAND domain of DEAF1 cause intellectual disability with severe speech impairment and behavioral problems. *Am. J. Hum. Genet.* **94**, 649–661 (2014).

27. Goldenberg, A. *et al.* Clinical and molecular findings in 39 patients with KBG syndrome caused by deletion or mutation of ANKRD11. *Am. J. Med. Genet. A* **170**, 2847–2859 (2016).

**Supplementary tables**

Sup Table 1. *De novo* PTVs among ASD patients from SSC, ASC, ACGC, TASC, MSSNG and AGRE cohorts.

Sup Table 2. List of gene with a significant excess of *de novo* truncating variants in the literature. DNM: de novo mutation, FDR: false discovery rate. o/e: observed/expected ratio of PTVs in the gnomAD dataset, LOEUF: Loss-of-function Observed/Expected Upper bound Fraction. Note that 3 genes (*NUDT17, NFE2L3* and *RANBP17*) were not retained because of an excessive o/e ratio.

Sup Table 3. List of 217 genes conferring ASD susceptibility prioritized in this study. R: recessive, D: dominant, X: X linked, NS: not scored.

Sup Table 4. CNVs of unknown significance found among the 253 ASD subjects. NA: not assessed, E: exon, BP: breakpoint, DN: de novo, Del: deletion, Dup: duplication, Trip: triplication, F: female, M: male, S: sporadic, Fa: familial

Sup Table 5. Nucleotide variants of unknown significance found among the 253 ASD subjects. F: female, M: male, S: sporadic, Fa: familial, DN: de novo, o/e: observed/expected ratio of PTVs in GnomAD, GnAD: allelic frequency in the GnomAD database.
